# Supplementary material for: Genome-Wide Identification and Expression Analysis under Abiotic Stress of BrAHL Genes in Brassica rapa
Source: Int J Mol Sci. 2023 Aug 4;24(15):12447. doi: 10.3390/ijms241512447 (PMC10420281; doi:10.3390/ijms241512447)
Supplement: Supplementary file 1 [file ijms-24-12447-s001.zip › ijms-2528992-supplementary.pdf]

Table S1.The information of *BrAHL* gene family

Gene name	Gene ID	Chromosome	pI	MW (Da)	Protein Length (aa)	Subcellular location	*A. thaliana ID*	*A. thaliana* name
Bra033121	*BrAHL01*	A02:16692793-16694938	10.17	32862.58	310	nuclear	*AT4G12080*	*AtAHL01*
Bra019359	*BrAHL02*	A03:24708369-24709819	9.32	34325.29	329	nuclear	*AT4G22770*	*AtAHL02*
Bra013646	*BrAHL03*	A01:6829636-6831096	9.65	32097.02	305	nuclear	*AT4G22770*	*AtAHL02*
Bra013886	*BrAHL04*	A01:8241250-8242870	5.22	40707.98	381	nuclear	*AT4G25320*	*AtAHL03*
Bra019174	*BrAHL05*	A03:25865465-25867125	5.34	42151.39	396	nuclear	*AT4G25320*	*AtAHL03*
Bra010070	*BrAHL06*	A06:19158797-19160803	8.64	42146.13	403	nuclear	*AT5G62260*	*AtAHL06*
Bra029164	*BrAHL07*	A03:6657405-6659182	5.54	44607.32	424	nuclear	*AT5G51590*	*AtAHL04*
Bra028265	*BrAHL08*	A01:19148654-19150878	5.34	45472.21	429	nuclear	*AT5G51590*	*AtAHL04*
Bra035899	*BrAHL09*	A09:3006503-3008422	8.76	41453.46	392	nuclear	*AT5G62260*	*AtAHL06*
Bra037321	*BrAHL10*	A09:1076739-1078309	9.17	33474.95	316	chloroplast	*AT4G00200*	*AtAHL07*
Bra027642	*BrAHL11*	A09:7184665-7186470	9.11	40033.75	381	nuclear	*AT1G63470*	*AtAHL05*
Bra022946	*BrAHL12*	A03:7895972-7897496	10.42	27045.57	262	nuclear	*AT2G33620*	*AtAHL10*
Bra029271	*BrAHL13*	A02:26207870-26209854	5.41	42359.33	401	nuclear	*AT5G62260*	*AtAHL06*
Bra027801	*BrAHL14*	A09:6057065-6058781	8.82	39273.1	375	nuclear	*AT1G63470*	*AtAHL05*
Bra022555	*BrAHL15*	A02:8971022-8973076	5.04	43880.3	419	nuclear	*AT5G51590*	*AtAHL04*
Bra021865	*BrAHL16*	A04:14931533-14933220	9.85	36261.5	350	nuclear	*AT2G33620*	*AtAHL10*
Bra007595	*BrAHL17*	A09:29942794-29944321	9.18	35632.65	341	nuclear	*AT3G61310*	*AtAHL11*
Bra039309	*BrAHL18*	A04:18660151-18661830	9.89	35906.36	341	nuclear	*AT2G45850*	*AtAHL09*
Bra013270	*BrAHL19*	A01:4636634-4638228	9.77	45203.2	426	nuclear	*AT4G17950*	*AtAHL13*
Bra021027	*BrAHL20*	A08:10395913-10397488	9.57	42310.63	410	nuclear	*AT4G17950*	*AtAHL13*
Bra025511	*BrAHL21*	A04:8231960-8233620	9.43	29263.74	277	nuclear	*AT1G63470*	*AtAHL05*
Bra014444	*BrAHL22*	A04:668944-670455	9.38	34927.2	333	nuclear	*AT3G61310*	*AtAHL11*
Bra017536	*BrAHL23*	A09:16169601-16171096	7.94	37721.84	353	nuclear	*AT5G46640*	*AtAHL08*
Bra040133	*BrAHL24*	A01:28422944-28424967	8.73	40127.3	383	nuclear	*AT3G04590*	*AtAHL14*
Bra003446	*BrAHL25*	A07:13228891-13230382	9.37	36312	339	nuclear	*AT2G45850*	*AtAHL09*
Bra025717	*BrAHL26*	A06:7456852-7458094	10.05	31188.96	303	nuclear	*AT4G17950*	*AtAHL13*
Bra025716	*BrAHL27*	A06:7454227-7455402	9.07	27162.12	269	nuclear, cytosol	*AT4G17950*	*AtAHL13*
Bra020612	*BrAHL28*	A02:24211831-24213989	7.89	37039.69	352	nuclear	*AT3G04590*	*AtAHL14*
Bra009999	*BrAHL29*	A06:18552239-18554524	8.73	38979.9	372	nuclear	*AT3G04590*	*AtAHL14*
Bra003217	*BrAHL30*	A07:11943288-11944184	5.62	31365.66	298	nuclear, peroxisome	*AT3G55560*	*AtAHL15*
Bra014735	*BrAHL31*	A04:2668587-2669510	5.77	32180.7	307	nuclear, chloroplast	*AT3G55560*	*AtAHL15*
Bra004732	*BrAHL32*	A05:1557907-1558692	9.4	27299.04	261	nuclear, chloroplast	*AT2G42940*	*AtAHL16*
Bra016853	*BrAHL33*	A04:17903238-17904023	9.6	27674.4	261	nuclear, chloroplast	*AT2G42940*	*AtAHL16*

Bra007176	*BrAHL34*	A09:27733461-27734354	5.28	31067.21	297	nuclear, chloroplast	*AT3G55560*	*AtAHL15*
Bra000281	*BrAHL35*	A03:10322727-10323512	9.4	27275.02	261	nuclear, chloroplast	*AT2G42940*	*AtAHL16*
Bra003717	*BrAHL36*	A07:14673382-14674245	6.53	29737.88	287	nuclear	*AT1G76500*	*AtAHL29*
Bra016444	*BrAHL37*	A08:18502443-18503390	6.56	32386.59	315	nuclear	*AT1G20900*	*AtAHL27*
Bra015738	*BrAHL38*	A07:21087029-21087895	6.54	29342.39	288	nuclear	*AT1G76500*	*AtAHL29*
Bra011612	*BrAHL39*	A01:1413633-1414541	6.22	30507.84	302	nuclear	*AT4G35390*	*AtAHL25*
Bra008262	*BrAHL40*	A02:14186272-14187135	6.38	29238.36	287	nuclear	*AT1G76500*	*AtAHL29*
Bra012256	*BrAHL41*	A07:9048236-9049165	6.56	31802.94	309	nuclear	*AT1G20900*	*AtAHL27*
Bra025854	*BrAHL42*	A06:8452918-8453852	6.63	29939.86	286	nuclear	*AT1G20900*	*AtAHL27*

Table S2.Analysis of homologous gene pairs in BrAHL gene family

gene-pairs	Ka	Ks	Ka/Ks	Selective type	Duplicated type
BrAHL01-BrAHL03	0.280489	0.954771	0.293776	Purifying	Segmental
BrAHL02-BrAHL03	0.0955655	0.241199	0.39621	Purifying	Segmental
BrAHL02-BrAHL05	0.520288	2.82633	0.184086	Purifying	Segmental
BrAHL02-BrAHL08	0.538111	2.54705	0.211268	Purifying	Segmental
BrAHL02-BrAHL10	0.443819	4.10755	0.10805	Purifying	Segmental
BrAHL02-BrAHL22	0.636944	4.15424	0.153324	Purifying	Segmental
BrAHL02-BrAHL32	0.959663	2.17172	0.441891	Purifying	Segmental
BrAHL02-BrAHL35	0.944031	3.1234	0.302245	Purifying	Segmental
BrAHL03-BrAHL10	0.434349	2.76188	0.157266	Purifying	Segmental
BrAHL03-BrAHL14	0.542935	4.10434	0.132283	Purifying	Segmental
BrAHL03-BrAHL23	0.73011	4.04681	0.180416	Purifying	Segmental
BrAHL03-BrAHL27	0.770983	3.96243	0.194573	Purifying	Segmental
BrAHL03-BrAHL32	0.870859	2.83658	0.30701	Purifying	Segmental
BrAHL04-BrAHL05	0.0794021	0.306141	0.259364	Purifying	Segmental
BrAHL04-BrAHL07	0.215247	0.791668	0.27189	Purifying	Segmental
BrAHL04-BrAHL09	0.480782	2.4852	0.193458	Purifying	Segmental
BrAHL04-BrAHL13	0.530591	2.27158	0.233578	Purifying	Segmental
BrAHL04-BrAHL15	0.253218	0.657761	0.38497	Purifying	Segmental
BrAHL05-BrAHL07	0.213512	0.756146	0.282369	Purifying	Segmental
BrAHL05-BrAHL08	0.206681	0.807694	0.25589	Purifying	Segmental
BrAHL05-BrAHL15	0.24878	0.683816	0.363811	Purifying	Segmental
BrAHL05-BrAHL37	0.76174	3.99483	0.190681	Purifying	Segmental

BrAHL06-BrAHL09	0.0695755	0.301692	0.230617	Purifying	Segmental
BrAHL06-BrAHL13	0.111439	0.284966	0.391062	Purifying	Segmental
BrAHL06-BrAHL23	0.770939	1.89216	0.407438	Purifying	Segmental
BrAHL07-BrAHL08	0.0688485	0.244546	0.281536	Purifying	Segmental
BrAHL07-BrAHL09	0.480059	2.20875	0.217344	Purifying	Segmental
BrAHL07-BrAHL10	0.453623	4.07888	0.111213	Purifying	Segmental
BrAHL07-BrAHL15	0.109213	0.239909	0.455226	Purifying	Segmental
BrAHL07-BrAHL27	0.758104	2.16899	0.349519	Purifying	Segmental
BrAHL08-BrAHL09	0.4999	2.91013	0.17178	Purifying	Segmental
BrAHL08-BrAHL15	0.10346	0.228465	0.452849	Purifying	Segmental
BrAHL09-BrAHL13	0.0997573	0.322037	0.309769	Purifying	Segmental
BrAHL09-BrAHL16	0.650114	3.4226	0.189948	Purifying	Segmental
BrAHL09-BrAHL18	0.673955	4.05521	0.166195	Purifying	Segmental
BrAHL09-BrAHL27	0.717451	3.95756	0.181286	Purifying	Segmental
BrAHL09-BrAHL31	0.839341	3.96812	0.211521	Purifying	Segmental
BrAHL09-BrAHL33	0.884043	3.91873	0.225594	Purifying	Segmental
BrAHL09-BrAHL34	0.755211	3.87664	0.194811	Purifying	Segmental
BrAHL09-BrAHL35	0.769351	2.56968	0.299396	Purifying	Segmental
BrAHL10-BrAHL14	0.594569	2.55929	0.232318	Purifying	Segmental
BrAHL10-BrAHL15	0.489911	4.08702	0.11987	Purifying	Segmental
BrAHL10-BrAHL21	0.605229	1.84627	0.327811	Purifying	Segmental
BrAHL10-BrAHL33	0.761789	3.87415	0.196634	Purifying	Segmental
BrAHL11-BrAHL14	0.0640308	0.254086	0.252004	Purifying	Segmental
BrAHL11-BrAHL17	0.527895	4.16217	0.126832	Purifying	Segmental
BrAHL11-BrAHL21	0.222999	1.12223	0.198711	Purifying	Segmental
BrAHL12-BrAHL16	0.119618	0.302093	0.395964	Purifying	Segmental
BrAHL12-BrAHL27	0.651365	1.99069	0.327206	Purifying	Segmental
BrAHL12-BrAHL42	0.972296	2.21217	0.439522	Purifying	Segmental
BrAHL14-BrAHL21	0.242218	1.08717	0.222797	Purifying	Segmental
BrAHL14-BrAHL25	0.584765	4.14052	0.14123	Purifying	Segmental
BrAHL14-BrAHL36	0.824912	4.00772	0.205831	Purifying	Segmental
BrAHL14-BrAHL38	0.782921	3.96999	0.19721	Purifying	Segmental
BrAHL16-BrAHL18	0.668807	3.29584	0.202925	Purifying	Segmental
BrAHL16-BrAHL20	0.513156	1.85878	0.276072	Purifying	Segmental
BrAHL16-BrAHL33	0.767302	3.70812	0.206925	Purifying	Segmental
BrAHL16-BrAHL36	0.896185	2.20434	0.406556	Purifying	Segmental
BrAHL17-BrAHL18	0.193032	1.5854	0.121756	Purifying	Segmental
BrAHL17-BrAHL22	0.0917582	0.366695	0.250231	Purifying	Segmental
BrAHL17-BrAHL25	0.153509	0.43531	0.352642	Purifying	Segmental
BrAHL17-BrAHL39	0.842052	2.40935	0.349493	Purifying	Segmental
BrAHL18-BrAHL22	0.229303	1.50783	0.152075	Purifying	Segmental
BrAHL18-BrAHL25	0.286845	2.12128	0.135223	Purifying	Segmental
BrAHL18-BrAHL32	0.914397	3.92714	0.23284	Purifying	Segmental
BrAHL18-BrAHL34	0.911426	4.00864	0.227365	Purifying	Segmental

BrAHL19-BrAHL20	0.212535	0.49884	0.426059	Purifying	Segmental
BrAHL19-BrAHL23	0.412677	1.61656	0.25528	Purifying	Segmental
BrAHL20-BrAHL23	0.282503	1.54362	0.183013	Purifying	Segmental
BrAHL20-BrAHL32	0.889119	3.95128	0.225021	Purifying	Segmental
BrAHL20-BrAHL33	0.820573	3.90137	0.210329	Purifying	Segmental
BrAHL20-BrAHL34	0.77319	3.93917	0.196282	Purifying	Segmental
BrAHL20-BrAHL37	0.763034	5.08463	0.150067	Purifying	Segmental
BrAHL22-BrAHL25	0.164855	0.581904	0.283303	Purifying	Segmental
BrAHL22-BrAHL34	0.894186	2.11766	0.422252	Purifying	Segmental
BrAHL23-BrAHL27	0.468204	1.2179	0.384434	Purifying	Segmental
BrAHL26-BrAHL27	0.160142	0.0707802	2.26253	positive	Tandem
BrAHL28-BrAHL29	0.0913436	0.319565	0.285837	Purifying	Segmental
BrAHL30-BrAHL31	0.0572405	0.283067	0.202216	Purifying	Segmental
BrAHL30-BrAHL33	0.536038	3.92501	0.13657	Purifying	Segmental
BrAHL33-BrAHL02	0.976039	1.92553	0.506893	Purifying	Segmental
BrAHL30-BrAHL34	0.0394178	0.332624	0.118506	Purifying	Segmental
BrAHL31-BrAHL34	0.0655846	0.321228	0.204168	Purifying	Segmental
BrAHL32-BrAHL33	0.0664934	0.389535	0.1707	Purifying	Segmental
BrAHL32-BrAHL35	0.0537802	0.284055	0.18933	Purifying	Segmental
BrAHL32-BrAHL36	0.556332	3.15053	0.176584	Purifying	Segmental
BrAHL33-BrAHL35	0.0748549	0.2804	0.266958	Purifying	Segmental
BrAHL36-BrAHL37	0.186638	1.02583	0.181938	Purifying	Segmental
BrAHL36-BrAHL38	0.0911994	0.316303	0.288329	Purifying	Segmental
BrAHL36-BrAHL39	0.412957	1.98022	0.208541	Purifying	Segmental
BrAHL36-BrAHL40	0.108539	0.233068	0.465695	Purifying	Segmental
BrAHL36-BrAHL41	0.213292	0.87377	0.244106	Purifying	Segmental
BrAHL36-BrAHL42	0.227232	1.06942	0.212481	Purifying	Segmental
BrAHL37-BrAHL38	0.153646	0.994577	0.154484	Purifying	Segmental
BrAHL37-BrAHL39	0.389795	4.1067	0.0949168	Purifying	Segmental
BrAHL37-BrAHL40	0.152198	1.07848	0.141122	Purifying	Segmental
BrAHL37-BrAHL41	0.060174	0.412277	0.145955	Purifying	Segmental
BrAHL37-BrAHL42	0.0436049	0.314245	0.138761	Purifying	Segmental
BrAHL38-BrAHL40	0.0915525	0.271385	0.337353	Purifying	Segmental
BrAHL38-BrAHL41	0.158667	0.789645	0.200934	Purifying	Segmental
BrAHL38-BrAHL42	0.181853	0.905378	0.200858	Purifying	Segmental
BrAHL39-BrAHL41	0.409692	4.09624	0.100017	Purifying	Segmental
BrAHL39-BrAHL42	0.383177	4.00286	0.0957258	Purifying	Segmental
BrAHL40-BrAHL41	0.189417	0.815105	0.232383	Purifying	Segmental
BrAHL40-BrAHL42	0.160445	0.941417	0.17043	Purifying	Segmental
BrAHL41-BrAHL42	0.0585612	0.324802	0.180298	Purifying	Segmental

Table S3. Primers used in this study

BrAHL01	qBr-033121-F	CCGTCAGATTTTCACATAGCTG
	qBr-033121-R	CACGTTTCTTCTTCATCGGTAC
BrAHL02	qBr-019359-F	CGAGGACGTAACGAAGAAGATA
	qBr-019359-R	GAGTCACTTGGCATGAATGATC
BrAHL04	qBr-013886-F	GTAGCAACTTTTATAGCGGCTC
	qBr-013886-R	GCTTTTCTTTCTTCTGCTGTGA
BrAHL17	qBr-007595-F	CTAGGAAGTACGGACAAGAAGG
	qBr-007595-R	CAATTCACCACTGGAACTAAGC
BrAHL18	qBr-039309-F	ATGCCTTCATCTTCTGGAATGA
	qBr-039309-R	CTAGCAGATAGAACGCAAATCG
BrAHL24	qBr-040133-F	TCTGAGCCGTTAAAGAGGAAAC
	qBr-040133-R	CTCTCTCCTCTCCTTAGCAGAG
BrAHL41	qBr-012256-F	AAACAAAGACCATCATCAACCG
	qBr-012256-R	TTGTTCTTAGATCCTGGTGGAC
BrAHL28	qBr-020612-F	CCAGTCAAGAGAAAGAGAGGTC
	qBr-020612-R	AACAATGTGCGGAGTAAAACTC
BrAHL29	qBr-009999-F	AGCAAAGCAAGCATGAGATATG
	qBr-009999-R	CGCTCCGAATATAAGATCCACT
BrAHL16	qBr-021865-F	GACCTCCTTCTTACACCGTTAG
	qBr-021865-R	GTCGTCCTCTCATCCTCTTAAC
BrACTIN2	qBrACTIN2-F	CGGTGTCAGGTTGGGATGA
	qBrACTIN2-R	CGTGCTCGATGGGGTACTTC
